# Effects of Pre-Anesthesia Anxiety on Propofol Induction Dose in Cats

**DOI:** 10.3390/ani11072126

**Published:** 2021-07-17

**Authors:** Yuki Shimizu, Teppei Kanda

**Affiliations:** 1 Veterinary Medical Teaching Hospital, Okayama University of Science, Imabari 794-8555, Ehime, Japan; y-shimizu@vet.ous.ac.jp; 2 Department Comparative Animal Science, Kurashiki University of Science and the Arts, Kurashiki 712-8505, Okayama, Japan; 3 Faculty of Veterinary Medicine, Okayama University of Science, Imabari 794-8555, Ehime, Japan

**Keywords:** cats, anxiety, anesthesia, animal welfare, veterinary science, veterinary nursing

## Abstract

**Simple Summary:**

In humans, the anxiety felt by patients before anesthesia reportedly increases the anesthetic requirements for anesthesia induction. Relieving the patient’s anxiety before anesthesia is necessary to prevent the excessive administration of anesthetic. However, no studies have been conducted on the effects of anxiety on anesthesia induction in cats. Therefore, we aimed to investigate the impact of the anxiety healthy cats felt before anesthesia on the propofol dose required for inducing anesthesia. We observed that significant behavioral changes due to anxiety did not increase the propofol dose requirements in the cats compared to those who experienced no anxiety. Furthermore, there was no difference in the maintenance and recovery from anesthesia between the two aforementioned conditions. In conclusion, anxiety due to changing circumstances did not change the propofol dose required for anesthesia induction in cats, despite significant changes in their behavior.

**Abstract:**

In humans, peri-anesthesia anxiety reportedly increases the anesthetic requirements for anesthesia induction. However, no studies have been conducted on cats regarding the effects of anxiety on anesthesia induction or anesthetic-mediated physiological changes. Therefore, we intended to investigate the effect of pre-anesthesia anxiety in healthy cats on the propofol dose required for anesthesia induction, and its impact on behavioral and physiological evaluations. The cats were placed in either a calm (CAL) or tense (ANX) environment. We performed physiological and behavioral evaluations before and after each environmental acclimatization period. Anesthesia was induced using propofol. We recorded the total dose of propofol administered for each clinical sign observed during anesthesia induction. The post-acclimatization behavioral evaluation score was significantly higher in the ANX group than the pre-acclimatization score. However, there was no significant difference in the propofol dose required for each clinical sign in the ANX or CAL groups. There were also no significant differences in the physiological evaluations between the ANX and CAL groups. Therefore, pre-anesthesia anxiety felt by cats did not affect propofol-mediated anesthesia induction.

## 1. Introduction

Cats brought to a veterinary clinic are often anxious, tense, and fearful. Cats are particularly anxious about environmental factors that differ from their daily environments, such as movement from a familiar environment to an unfamiliar one and the sounds or odors in animal hospitals. Anxiety, tension, and fear in cats can cause physiological changes, irrespective of the presence or absence of illness [1,2].

In human medicine, patient anxiety is of particular significance. Compared with adults, children are more likely to experience anxiety due to various factors. To reduce anxiety in children, their wards are generally accompanied by playrooms and wall decorations [3]. Furthermore, active efforts are made not just in the ward but also for the children’s perioperative or peri-anesthesia conditions. Appropriate intervention to reduce anxiety to the maximum possible extent in perioperative patients may result in sedative effects that are comparable to those observed with sedatives [4]. One study evaluated perioperative anxiety using the State-Trait Anxiety Scale (STAI) in patients who required surgery under general anesthesia [5,6]. It was observed that perioperative anxiety increased the propofol dose that was required to achieve a certain depth of anesthesia. Additionally, it affected the degree of pain and recovery process following surgery [5,6].

Another study that used the STAI to assess patient anxiety during the perioperative period and examined the propofol dose required to achieve a certain depth of anesthesia [7] showed that state and trait anxiety during the perioperative period did not affect the propofol concentration requirements [7]. Thus, the relationship between perioperative anxiety and the required propofol dose remains debatable [7].

It is important to reduce anxiety in patients as much as possible during the pre-anesthesia period, as anxiety and tension affect the patient’s condition during the perioperative period [8]. Efforts to reduce anxiety in patients during the perioperative period are considered an essential part of perioperative nursing; nurses create a safe environment for patients and communicate with them to reassure them about their treatment. However, no studies have reported on the effects of anxiety, tension, and fear on the anesthesia procedure and anesthetic-mediated physiological changes in cats.

In veterinary medicine, the American Association of Feline Practitioners and the International Society of Feline Medicine (ISFM) have established guidelines for the safer treatment of cats [9,10]. These guidelines describe the procedures for handling cats in veterinary clinics. In addition, they describe the kind of environment that should be prepared in veterinary hospitals. Based on these guidelines, the ISFM has proposed idea of the “Cat-Friendly Clinic” to provide safe, secure, and appropriate veterinary care to cats and their owners. The idea of a Cat-Friendly Clinic has recently become popular worldwide and refers to a veterinary clinic that takes the unique needs of cats into consideration.

The number of Cat Friendly Clinics has recently increased and become widespread. Moreover, protecting the health and welfare of cats has been recognized as an important issue. Therefore, veterinary hospitals have recognized the importance of considering anxiety, tension, and fear in cats.

We aimed to investigate the impact of pre-anesthesia anxiety in healthy cats on the dose of propofol required for inducing anesthesia and its effect on behavioral and physiological evaluations of the cats.

## 2. Materials and Methods

### 2.1. Animals

Six purpose-bred clinically healthy cats (three neutered females, one intact, and two neutered males), aged 65.5 (14–96) months (median (range)) and weighing approximately 3.9 (3.0–4.4) kg were used in this study. All cats were housed under uniform conditions at an institutional animal laboratory. A 12:12 h light–dark cycle (light period, 8:00 a.m.–8:00 p.m.) was maintained, and the room temperature and humidity was maintained at 23 ± 1 °C and 50 ± 20%, respectively. Each cat was used twice for each treatment, with a washout period of at least 1 week. The cats were fasted for 15 h before beginning the study and were provided free access to water during the 1 h before the commencement of the study. All procedures were approved by the Animal Research Committee of Kurashiki University of Science and the Arts, Okayama, Japan (No. 29-05).

### 2.2. Procedures

We conducted behavioral and physiological evaluations in the animal laboratory before beginning each experiment in all of the cats. The cats were moved to the treatment room, and their body weight was measured. We placed a 24-gauge/25-mm catheter (Gelco I.V. Catheter II, Smiths Medical Japan Co., Ltd., Tokyo, Japan) into the cephalic vein of each cat. They were acclimatized to the environment for 60 min. Following acclimatization, we conducted the physiological and behavioral evaluations in each environment.

The cats were then moved to the treatment room within 1 min. They were premedicated with 20 µg/kg intravenous atropine (0.5 mg atropine sulfate injection “Fuso”, Alfresa Pharma Co., Ltd., Osaka, Japan) before anesthesia induction. General anesthesia induction was performed with propofol (1% 100 mL intravenous propofol injection “FK,” Fresenius Kabi Japan Co., Ltd. mg/kg) at 8 mg/kg/5 min (1.6 mg/kg/min) using an infuser (Top Animal Syringe Pump TOP-551VC, Top Co., Ltd., Tokyo, Japan). Moreover, we recorded the total dose of propofol administered for each clinical sign observed during induction. The cats were intubated following the disappearance of the laryngeal reflex. Lidocaine lubricant gel was applied to the tip of the endotracheal tube; however, no local anesthetics were applied to the larynx before intubation.

The anesthesia was maintained for 60 min from the beginning (T0) to the end of inhalation anesthetic administration (T60). Anesthesia was maintained with isoflurane (isoflurane for animals, Zoetis Japan Co., Ltd., Tokyo, Japan), oxygen, and an anesthesia machine (anesthetic machines for animals, Acoma Medical Industry Co., Ltd., Tokyo, Japan). We terminated the anesthetic administration 60 min after commencing anesthesia maintenance.

We performed extubation after the onset of spontaneous respiration or recovery of the laryngeal reflex. At the time of awakening, the time and quality of recovery from anesthesia were evaluated. We continued to monitor the cats until they could keep their heads up.

**Figure 1:**
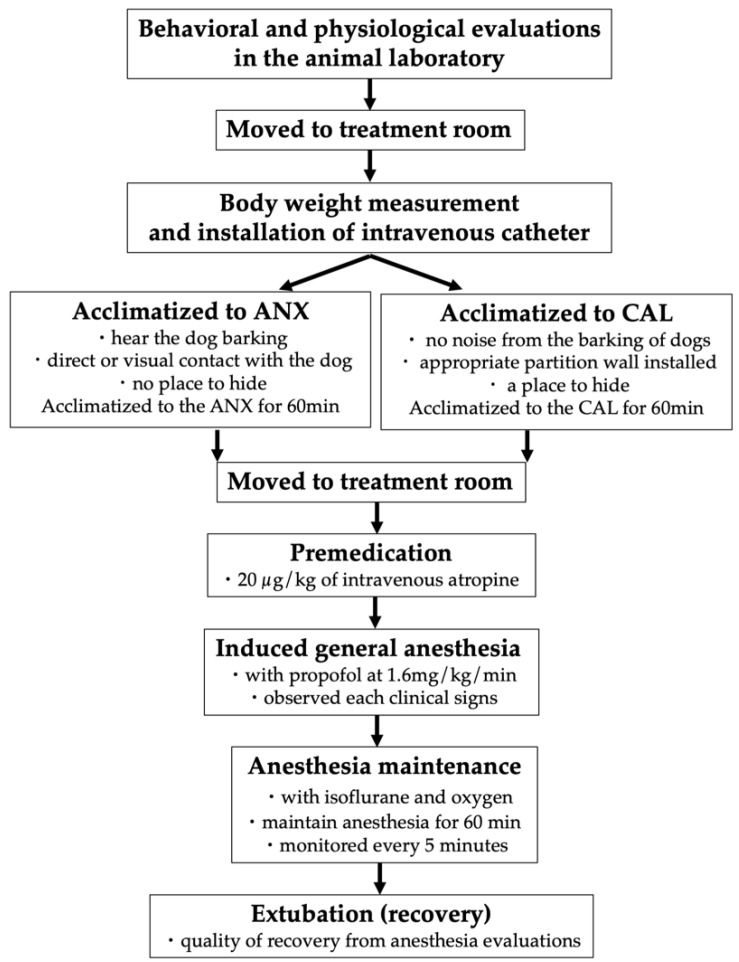
Experimental procedure of the study.

We performed a physiological evaluation the following day. After confirming no problem in the general condition of the cats, we terminated the experiment. The process of the series of experimental procedures is shown in Figure 1.

### 2.3. Anesthetic Management

Anesthesia was maintained by the inhalation anesthetic, such that the exhaled isoflurane concentration was 2.0%. During anesthesia maintenance, we used an animal biometric information monitor (“animal” multipurpose monitor bioscope AM130 (B), Fukuda ME Kogyo Co., Ltd., Chiba, Japan) and an oscillometric sphygmomanometer (animal sphygmomanometer Pet Map, Co., Ltd., Tokyo, Japan) for monitoring.

The heart rate (HR), electrocardiogram, pulse rate (PR), non-invasive blood pressure (BP), hemoglobin oxygen saturation, rectal temperature (RT), end-tidal isoflurane concentration, and end-tidal partial pressure of carbon dioxide were monitored from T0 to T60 every 5 min. Moreover, we reduced the exhaled isoflurane concentration by 0.2% when the mean blood pressure (MAP) dropped below 65 mmHg until it recovered to 65 mmHg.

We recorded the total time during which the MAP fell below 65 mmHg. Respiratory management was performed during spontaneous breathing. In addition, we performed respiratory assistance if the end-tidal carbon dioxide concentration deviated from the range of 35–45 mmHg.

### 2.4. Physiological Evaluation

For physiological evaluation, the systolic (SAP), diastolic (DAP), and MAP (mmHg) were measured using an oscillometric sphygmomanometer (Pet Map for animals, AVS, Tokyo, Japan). We measured the rectal temperature (°C) using an electronic rectal thermometer for animals (Thermo Flex for Animals, Astec, Ibaraki, Japan). The heart rate (beats/min) was measured by thoracic auscultation, and the pulse rate (beats/min) was measured by a palpation of the femoral artery. Moreover, the respiratory rate (RR; beats/min) was measured by the movement of the thorax.

### 2.5. Environmental Treatments

We set up two environment groups to acclimatize the cats. Each cat that was expected to feel anxiety or fear was placed under three conditions as follows: that cat could hear a dog barking, had direct or visual contact with dogs, and had no place to hide (ANX group). To achieve this, a cage was installed in the kennel space.

Each cat that was expected to not feel anxiety or fear was placed under three conditions as follows: no noise from barking dogs, appropriate partition walls were installed, and there was a place to hide (CAL group). To achieve this, the cage was installed in an ordinary animal laboratory.

All of the cats were subjected to the two aforementioned conditions during the study period. The order of the environments the cats were placed in was ANX–CAL and CAL–ANX, which each group consisting of three cats.

### 2.6. Behavioral Evaluation

The behavioral evaluation method used in this study was a modification of the method used in previous reports [11,12]. For the behavioral evaluation, 11 parameters of posture, body, belly, legs, head, tail, eyes, pupils, ears, whiskers, vocalization, and activity were observed (Table A1). All parameters were evaluated on a scale of 4–5, and the total score was calculated. The evaluators were unified throughout all experiments (Y.S.). Moreover, the anesthesia practitioner (T.K.) was not informed of the environment to which the test cat was adapted. The behavioral evaluation was performed within 2 min throughout all experiments.

### 2.7. Clinical Signs Observation

The depth of anesthesia was assessed by observing clinical signs. The clinical signs evaluated comprised loss of body movement; appearance of the nictitating membrane; disappearance of the eyelid reflex, flexion reflex, and tongue retraction reflex; loss of mandibular tension; and disappearance of the corneal reflex and laryngeal reflex. We recorded the dose of propofol administered for each clinical sign.

### 2.8. The Quality of Recovery

Based on the methods by Kennedy et al. and Mathis et al., the quality of recovery was evaluated on a 4-point scale as follows: very good (no excitement, paddling, vocalizing, trembling, vomiting, or convulsions), good (little excitement, no paddling, vocalizing, trembling, vomiting, or convulsions), slightly poor (excitement, some paddling, vocalizing, trembling, vomiting, no convulsions), and poor (extreme excitement, aggression, vocalizing, violent movement or convulsions, intervention necessary) [13,14].

### 2.9. Statistical Analyses

All statistical analyses were performed using a statistical analysis software (GraphPad Prism 6.0, GraphPad Software, Inc., San Diego, CA, USA). We performed the Wilcoxon test to compare the behavioral and physiological evaluations (BP, SAP, MAP, DAP, HR, PR, and RR) before and after acclimatization in both groups at similar time points. It was used to compare the HR, BP, and RR during anesthesia maintenance; the propofol dose for each clinical sign observed during induction; the time required before the cat could keep its head up; and the recovery scores between the groups. Moreover, we performed the Friedman test for repeated measurements using Dunn’s multiple comparison test as the post-test to compare the HR and BP between the ANX and CAL groups during anesthesia maintenance. A Spearman’s rank correlation test was conducted to examine the correlations of the propofol dose with the behavioral evaluation score and the BP. Statistical significance was set at *p* < 0.05.

## 3. Results

### 3.1. Environmental Acclimatization

#### 3.1.1. Behavioral Evaluation

**Figure 2:**
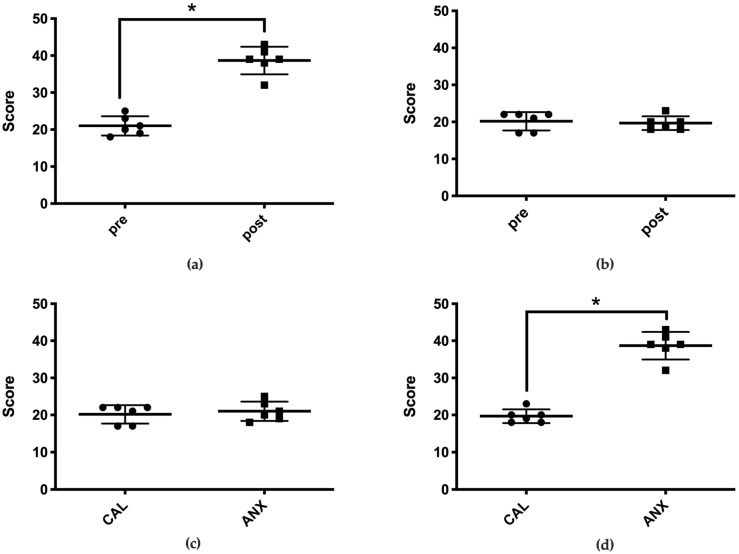
Each circle and square indicate the total behavioral evaluation score of each cat. The thick horizontal lines and error bars represent the six medians and ranges. The “pre” on the horizontal axis denotes the total behavioral evaluation score before environmental acclimatization, and the “post” denotes the total score after environmental acclimatization. “CAL” and “ANX” on the horizontal axis refer to their respective groups. * Indicates statistical significance. The graphs depict the following: (**a**) the pre- and post-environmental acclimatization behavioral evaluation scores in the ANX group; (**b**) the pre- and post-environmental acclimatization behavioral evaluation scores in the CAL group; (**c**) the pre-environmental acclimatization behavioral evaluation scores in both groups; (**d**) the post-environmental acclimatization behavioral evaluation scores in both groups. * Significantly different between groups (*p* < 0.05).

In the ANX group, the behavioral evaluation score following acclimatization was 39 (32–43) points (median (range)), which was significantly higher than the pre-acclimatization score of 20 (18–25) points (Figure 2a). However, no significant difference was observed between the pre- (21 (17–22)) and post-acclimatization (19 (18–23)) behavioral scores in the CAL group (Figure 2b). The post-acclimatization behavioral score in the ANX group was significantly higher than that of the CAL group (Figure 2c,d).

#### 3.1.2. Physiological Evaluation

There were no statistically significant differences in the HR, PR, RR, and BP (SAP, MAP, and DAP) between the pre- and post-acclimatization periods in both groups.

### 3.2. Anesthesia Induction

#### 3.2.1. Propofol Dose Required to Observe Each Clinical Sign

There were no statistically significant differences in the propofol dose required to observe each clinical sign between the groups, namely the loss of body movement, the appearance of the nictitating membrane, the loss of the eyelid reflex, the disappearance of the flexion reflex and the tongue retracting reflex, the loss of mandibular tension, and the disappearance of the corneal and laryngeal reflex (Table 1).

#### 3.2.2. Behavioral Evaluation Score and Propofol Requirements

**Figure 3:**
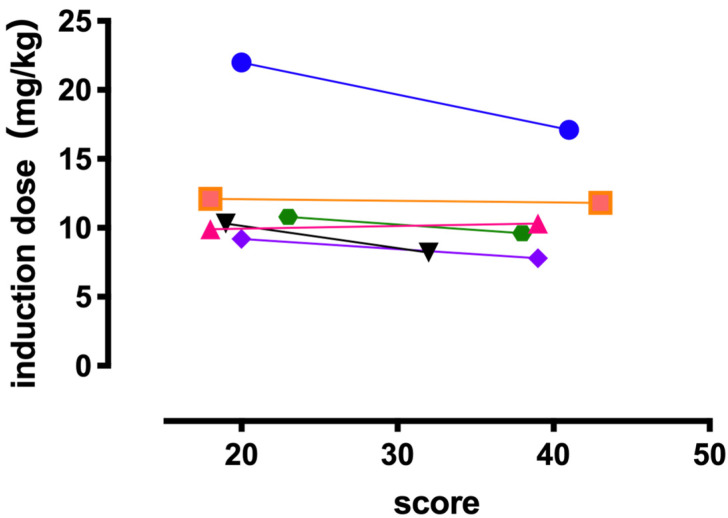
Each circle indicates the total behavioral evaluation score of each cat. It shows the correlation between the behavioral evaluation score plotted on the horizontal axis and the propofol dose requirement until the completion of endotracheal intubation plotted on the vertical axis.

We observed no significant correlation between the behavioral evaluation score following acclimatization and the dose of propofol required for completing endotracheal intubation in both groups (Figure 3).

### 3.3. Anesthesia Maintenance

#### 3.3.1. Heart Rate

There were no significant changes in the HR from T5 to T60 within the groups, compared to that at T0 (ANX group, 174 ± 16 beats/min; CAL group, 153 ± 7 beats/min). No significant difference was observed between the groups at any time point while comparing the HR every 5 min (Table 2).

#### 3.3.2. Blood Pressure

The SAP decreased significantly from T5 to T30 within the ANX group (92 ± 11 mmHg, 93 ± 6 mmHg, 93 ± 5 mmHg, 101 ± 6 mmHg, 101 ± 5 mmHg, and 102 ± 4 mmHg) and from T5 to T15 in the CAL group (89 ± 5 mmHg, 92 ± 4 mmHg, and 97 ± 5 mmHg), compared to that at T0 (ANX group, 158 ± 12 mmHg; CAL group, 155 ± 12 mmHg) (Table 2). The MAP decreased significantly from T5 to T25 within the ANX group (66 ± 8 mmHg, 64 ± 5 mmHg, 64 ± 4 mmHg, 67 ± 3 mmHg, and 69 ± 2 mmHg) and from T5 to T25 and at T40 within the CAL group (64 ± 5 mmHg, 66 ± 3 mmHg, 69 ± 3 mmHg, 71 ± 2 mmHg, 72 ± 2 mmHg, and 74 ± 3 mmHg), compared to that at T0 (ANX group, 134 ± 9 mmHg; CAL group, 129 ± 8 mmHg). The DAP decreased significantly from T5 to T25 within the ANX group (52 ± 7 mmHg, 48 ± 4 mmHg, 48 ± 3 mmHg, 50 ± 3 mmHg, and 51 ± 3 mmHg), and from T5 to T25 in the CAL group (47 ± 2 mmHg, 51 ± 3 mmHg, 53 ± 3 mmHg, 53 ± 3 mmHg, and 56 ± 3 mmHg), compared to that at T0 (ANX group, 113 ± 7 mmHg; CAL group, 114 ± 7 mmHg).

Comparison of the SAP, MAP, and DAP every 5 min between the ANX and CAL groups demonstrated no significant differences at any time point. The MAP fell below 65 mmHg for 7.5 min (0–25 min) (median (range)) and 12.5 min (0–40 min) in the ANX and CAL groups, respectively. The total time in the CAL group tended to be slightly longer when the MAP was <65 mmHg. Nonetheless, there was no significant difference at any time point.

#### 3.3.3. Respiratory Rate

There were no significant changes in the RR from T5 to T60 within the groups, compared to that at T0 (ANX group, 12 ± 4 beats/min; CAL group, 10 ± 3 beats/min). No significant difference was observed between the groups at any time point when comparing the RR every 5 min.

### 3.4. The Time during Which the MAP Was <65 mmHg and the Propofol Dose Required

There was no significant correlation between the propofol dose required to complete endotracheal intubation and the time during which the MAP was below 65 mmHg in both groups.

### 3.5. Recovery

#### 3.5.1. Time to Head Up (HUT: Head Up Time)

The cats in the ANX and CAL groups required 32.5 (12.0–39.0) min (median [range]) and 25.5 (12–42) min, respectively, to keep their heads up after the end of inhalation anesthetic administration. No significant difference was observed in the HUT between the groups.

#### 3.5.2. The Quality of Recovery

The evaluation score of the quality of recovery was 1.0 point, suggesting good quality in all cats in both groups. No significant differences were observed in the evaluation score of the recovery quality between the groups.

## 4. Discussion

The behavioral evaluation score in the ANX group increased significantly following an exposure to anxious and fearful circumstances. However, it did not change in the CAL group that was exposed to ordinary circumstances. The score in the ANX group following exposure was significantly higher than that in the CAL group. Therefore, the cats in the ANX group experienced anxiety or fear that was enough to induce behavioral changes.

In humans, anxiety gets transmitted to the central nervous system through the sensory organs, causing an activation of the hypothalamus-anterior pituitary–adrenal cortex system or an activation of the sympathetic and adrenal medullary systems [15,16,17,18]. Thus, increased cardiac output and vasoconstriction-related increased peripheral vascular resistance elevate the BP.

In contrast, no significant changes in the HR, PR, SAP, MAP, and DAP were observed in the cats following an exposure to each circumstance in either group. Specifically, anxiety or fear felt by the cats did not induce any changes in their physiological functions, such as the HR, PR, and BP. Furthermore, in this study, the time to acclimatize to each environment was 60 min. It is possible that the anxiety felt by the cat was inadequate in terms of duration and strength to affect the HR, PR, and BP. Further, it has been indicated in a human study that a patient in whom significant anxiety was detected through subjective methods did not show any physiological responses, including changes in HR, i.e., there might be no significant relationship between psychological and physiological measures of anxiety [19,20]. The RR did not demonstrate a specific trend in either group. In a previous study, the RR was included in the behavioral evaluation rather than a physiological one [11]. However, despite statistically significant fluctuations in the RR, their clinical relevance to the fluctuations varies. This, in turn, makes it difficult to evaluate anxiety or fear in cats [1]. Therefore, we were unable to further discuss the effects of anxiety on the RR in cats.

There was no significant difference in the propofol dose required to obtain each clinical sign observed during anesthesia induction and to complete endotracheal intubation between the groups. In addition, there was no correlation between the behavioral evaluation scores and the propofol dose required for endotracheal intubation in any group. Specifically, anxiety or fear felt by the cats due to the surrounding circumstances did not affect the induction dose of propofol. In humans, a greater degree of anxiety before anesthesia is associated with a higher propofol dose required to reach a certain depth of anesthesia [5,6]. Kil suggested that patients with significant anxiety experience increased subjective feelings of worry and apprehension and more dramatic neuroendocrine responses to stimuli, thereby resulting in greater anesthetic requirements during surgery. However, researchers have not yet investigated the relation between increased anxiety and a greater dose of anesthetics in humans.

Human anxiety is classified into the following two types: (i) trait anxiety, which refers to an individual’s original personality anxiety that does not fluctuate depending on the situation and (ii) state anxiety, which refers to anxiety that fluctuates depending on the situation and is felt transiently. Strong trait anxiety not only increases the required anesthetic dose but also increases pre-anesthetic state anxiety [6]. The anxiety felt by the cats was induced transiently, owing to their direct or visual contact with the dog and no place to hide. It did not increase the required propofol dose. Thus, the anxiety felt by the cats might correspond to state anxiety rather than trait anxiety in humans. However, there are no reports on the categorization of anxiety in cats in the way that there are for humans. Therefore, we could not determine if the anxiety observed in this study was state anxiety. Moreover, irrespective of the type of anxiety, the induction dose of propofol for the cats was not affected in this study.

Furthermore, regardless of the anxiety felt by the cat before inducing anesthesia, there were no significant differences in the HR and BP between the ANX and CAL groups during anesthesia maintenance. Moreover, transition tendencies in both HR and BP were similar in both groups. For a higher propofol dose requirement in either group, the BP was likely to be lower than that in the other group, particularly in the early phase of anesthesia maintenance. Propofol decreased the cardiac output and total peripheral vascular resistance, resulting in a dose-dependent decrease in the arterial BP. HR fluctuations due to propofol are rare; however, marked hypotension is often observed [21]. Further, the blood levels of propofol drop rapidly due to its rapid redistribution and metabolism. In addition, its action duration is estimated to be 10–40 min [21]. We observed no statistical differences between the groups regarding these factors. The reason for no differences observed in the cardiovascular functions such as the HR and arterial BP during anesthesia maintenance might have been the unchanged propofol dose requirement despite the anxiety the cats felt. There was no significant difference in the HUT between the CAL and ANX groups. Therefore, the anxiety felt by the cats did not affect the HUT after terminating inhalation anesthetic administration.

There was no significant difference in the recovery quality between the ANX and CAL groups. There are no reports on the impact of pre-anesthesia anxiety on the quality of recovery in humans or cats. Our findings suggest that the pre-anesthesia anxiety felt by the cats did not affect the quality of recovery from general anesthesia.

The anxiety felt by the cats did not affect anesthesia induction using propofol. It is desirable to reduce the anxiety experienced by cats during the peri-anesthesia period. However, it is extremely difficult to completely eliminate such anxiety in veterinary hospitals. Greater than necessary doses of propofol should not be administered to induce anesthesia in cats who show no physiological effects but are clearly anxious.

We should acknowledge some limitations in this study. First, we did not choose the cats based on their sex, age variations, or whether they were accustomed to the environment or not, which might have affected the results since all the cats housed at our animal laboratory at that time were used in this study. Second, HR and arterial BP observed during anesthesia might have been increased due to atropine administration. These changes might mask the intra- or inter-group differences in the HR and BP. Finally, the small sample size of animals made it impossible to exclude type II errors due to the low detection power (1 − β).

## 5. Conclusions

We conclude that anxiety in cats, which induced behavioral changes but not physiological ones due to the effects of the condition, did not affect the propofol dose required to induce anesthesia in the cats. It also had no effects on the cardiovascular functions during anesthesia maintenance. This impact of anxiety on the anesthetic dose required in cats was a novel finding of our study. Moreover, anxiety exerted no effects on the time-to-head-up or quality of recovery after terminating anesthetic administration, compared to cats with less anxiety. In terms of clinical relevance, despite substantial anxiety in cats, a larger dose of propofol would not be required to induce general anesthesia.

## Figures and Tables

**Table 1:** Propofol dose required to observe each clinical sign during anesthesia induction.

Clinical Sign	Propofol Dose Required in the ANX Group (Median [Range])	Propofol Dose Required in the CAL Group (Median [Range])
Loss of body movement	3.1 mg/kg (2.2–4.0 mg/kg)	4.0 mg/kg (2.4–6.9 mg/kg)
Appearance of nictitating membrane	3.2 mg/kg (2.3–6.1 mg/kg)	3.1 mg/kg (1.6–4.4 mg/kg)
Loss of eyelid reflex	5.4 mg/kg (2.2–9.6 mg/kg)	5.4 mg/kg (2.4–9.9 mg/kg)
Disappearance of flexion reflex	6.9 mg/kg (5.0–9.1 mg/kg)	7.5 mg/kg (4.3–8.5 mg/kg)
Disappearance of tongue retracting reflex	5.6 mg/kg (3.9–6.6 mg/kg)	6.4 mg/kg (4.4–7.4 mg/kg)
Loss of mandibular tension	5.4 mg/kg (3.9–6.6 mg/kg)	6.3 mg/kg (3.8–7.4 mg/kg)
Disappearance of corneal reflex	9.9 mg/kg (6.2–12.2 mg/kg)	7.0 mg/kg (4.4–11.6 mg/kg)
Disappearance of laryngeal reflex	9.9 mg/kg (7.8–17.1 mg/kg)	10.5 mg/kg (9.2–22.0 mg/kg)

Propofol dose required to observe each clinical sign during anesthesia induction in the ANX and the CAL groups.

**Table 2:** The value of HR and MAP at each measurement time.

HR and MAP	Each Measurement Time during Maintenance of Anesthesia						
	**T0**	**T5**	**T10**	**T15**	**T20**	**T25**	**T30**
HR (beats/min)							
ANX	174 ± 16	166 ± 7	158 ± 6	152 ± 5	150 ± 5	152 ± 5	152 ± 5
CAL	153 ± 7	153 ± 11	142 ± 11	142 ± 8	144 ± 7	146 ± 6	145 ± 6
MAP (mmHg)							
ANX	134 ± 9	66 ± 8	64 ± 5	64 ± 4	67 ± 3	69 ± 2	71 ± 3
CAL	129 ± 8	64 ± 5	66 ± 3	69 ± 3	71 ± 2	72 ± 2	77 ± 2
	**T35**	**T40**	**T45**	**T50**	**T55**	**T60**	
HR (beats/min)							
ANX	154 ± 5	153 ± 6	151 ± 6	148 ± 6	146 ± 5	145 ± 5	
CAL	148 ± 6	147 ± 7	144 ± 7	144 ± 7	143 ± 7	143 ± 7	
MAP (mmHg)							
ANX	79 ± 5	79 ± 2	76 ± 2	75 ± 3	72 ± 3	69 ± 4	
CAL	78 ± 3	74 ± 3	77 ± 3	78 ± 3	76 ± 4	76 ± 2	

## Data Availability

Not applicable.

## Appendix A

**Table A1:** Behavioral evaluation.

Observation	Description	Score
Body	Laid out on side or on back: (I)	1
	NA: (A)	1
	Laid ventral or half on side: (I)	2
	Standing or moving, back horizontal: (A)	2
	Laid ventral or sitting: (I)	3
	Standing or moving: (A)	3
	Laid ventral, rolled, or sitting: (I)	4
	Standing or moving, body behind lower than in front: (A)	4
	laid ventrally or crouched directly on top of all paws, may be shaking: (I)	5
	whole body near to ground, crawling, may be shaking: (A)	5
Belly	Exposed, slow ventilation	1
	Exposed or not exposed, slow or normal ventilation	2
	Not exposed, normal ventilation	3
	Not exposed, normal or fast ventilation	4
Legs	Fully extended: (I)	1
	NA: (A)	1
	Bent, hind legs may be laid out: (I)	2
	when standing extended: (A)	2
	Bent: (I)	3
	While standing extended: (A)	3
	Bent: (I)	4
	When standing hind legs bent, in front extended: (A)	4
	Bent: (I)	5
	Bent near the surface: (A)	5
Head	Laid on the surface with chin upwards or on the surface	1
	Laid on the surface or over the body, some movement	2
	Over the body or pressed to the body, little or no movement	3
	On the plane of the body, less or no movement	4
	Lower than the body, motionless	5
Pupils	Normal (consider ambient light)	1
	Normal or partially dilated	2
	Dilated	3
	Fully dilated	4
Ears	Normal (half back)	1
	Normal (half back) or erect and moved to the front	2
	Normal (half back) or erect and moved to the front or back and forward on the head	3
	Partially flattened	4
	Fully flattened back on the head	5
Whiskers	Normal (lateral)	1
	Normal (lateral or forward)	2
	Lateral (normal) or forward and back	3
	Back	4
Tail	Extended or loosely wrapped: (I)	1
	NA: (A)	1
	Extended or loosely wrapped: (I)	2
	Tail up or loosely downwards: (A)	2
	On the body or curved backwards, may be twitching: (I)	3
	Up or tense downwards, may be twitching: (A)	3
	Close to the body: (I)	4
	Tense downwards or curled forward, may be twitching: (A)	4
	Close to the body: (I)	5
	Curled forward close to the body: (A)	5
Eyes	Closed or half open, may be slowly blinking	1
	Closed, half open or completely, normally open	2
	Normally open	3
	Widely open	4
	Completely open	5
Vocalization	None or soft purr	1
	None or Meow	2
	Meow, plaintive meow, or quiet	3
	Plaintive meow, yowling, growling, or quiet	4
	Plaintive meow, yowling, growling, hissing, or quiet	5
Activity	Sleeping or resting	1
	Sleeping, resting, alert or active, or may be playing	2
	Resting, awake, or actively exploring	3
	Alert, may be actively trying to escape, motionless alert, or actively prowling	4
	Immovable	5

(I) indicates inactive cats. (A) indicates active cats. NA indicates that not applicable.
